# Modified indirect calorimetry for patients on venoarterial extracorporeal membrane oxygenation: a pilot feasibility study

**DOI:** 10.1038/s41430-023-01291-x

**Published:** 2023-05-17

**Authors:** Oana A. Tatucu-Babet, Arne Diehl, Caroline Kratzing, Kate Lambell, Aidan Burrell, Audrey Tierney, Ibolya Nyulasi, Michael Bailey, Jayne Sheldrake, Emma J. Ridley

**Affiliations:** 1 Australian and New Zealand Intensive Care Research Centre, School of Public Health and Preventive Medicine, Monash University, Melbourne, VIC Australia; 2 Nutrition Department, The Alfred, Melbourne, VIC Australia; 3 Department of Intensive Care and Hyperbaric Medicine, The Alfred, Melbourne, VIC Australia; 4 School of Allied Health and Health Implementation Science and Technology Centre, Health Research Institute, University of Limerick, Limerick, Ireland; 5 Department of Dietetics, Nutrition and Sport, La Trobe University, Melbourne, VIC Australia; 6 Department of Medicine, Central Clinical School, Monash University, Melbourne, VIC Australia

**Keywords:** Diseases, Medical research

## Abstract

**Background/Objectives:**

Traditional indirect calorimetry is unable to capture complete gas exchange in patients receiving venoarterial extracorporeal membrane oxygenation (VA ECMO). We aimed to determine the feasibility of using a modified indirect calorimetry protocol in patients receiving VA ECMO, report measured energy expenditure (EE) and compare EE to control critically ill patients.

**Subjects/Methods:**

Mechanically ventilated adult patients receiving VA ECMO were included. EE was measured within 72 h of VA ECMO commencement (timepoint one [T1]) and on approximately day seven of Intensive Care Unit (ICU) admission (timepoint two [T2]). Traditional indirect calorimetry via the ventilator was combined with calculations of oxygen consumption and carbon dioxide production derived from pre- and post-ECMO membrane blood gas analyses. Completion of ≥60% EE measurements was deemed feasible. Measured EE was compared between T1 and T2 and to control patients not receiving VA ECMO. Data is presented as *n*(%) and median[interquartile range (IQR)].

**Results:**

Twenty-one patients were recruited; 16(76%) male, aged 55[42–64] years. The protocol was feasible to complete at T1 (14(67%)) but not at T2 (7(33%)) due to predominantly ECMO decannulation, extubation or death. EE was 1454[1213–1860] at T1 and 1657[1570–2074] kcal/d at T2 (*P* = 0.043). In patients receiving VA ECMO versus controls, EE was 1577[1434–1801] versus 2092[1609–2272] kcal/d, respectively (*P* = 0.056).

**Conclusion:**

Modified indirect calorimetry is feasible early in admission to ICU but is not possible in all patients receiving VA ECMO, especially later in admission. EE increases during the first week of ICU admission but may be lower than EE in control critically ill patients.

## Introduction

Extracorporeal membrane oxygenation (ECMO) is a specialised organ support for critically ill patients with severe respiratory and/or cardiac failure. Deoxygenated blood is passed through an external gas exchange system before being returned to the circulation [1]. Extracorporeal membrane oxygenation can be described as either venoarterial (VA) or venovenous (VV), depending on the site where oxygenated blood is returned [1]. Previously, it was presumed that ECMO may increase energy expenditure (EE) and protein catabolism, in part due to the associated inflammatory response [2–5]. The mode of ECMO may also alter important clinical factors related to metabolism during critical illness. In VV ECMO, a recent study reported a decrease in inflammation and in a second, measured EE was comparable to matched critically ill patients not receiving VV ECMO [6, 7]. Conversely, very little is known regarding EE during VA ECMO which is indicated in an entirely different patient cohort with cardiogenic shock, where part of the cardiac work is relieved by the mechanical pump [8].

Predictive equations are the most common method of determining EE in critical illness but are known to significantly under- or overestimate requirements compared to measured EE using indirect calorimetry [9]. Accurate determination of EE is important in patients receiving ECMO who typically have higher illness severity and longer hospital admissions, making them more vulnerable to under- and overfeeding over the course of ICU admission [3, 10]. Indirect calorimetry is the current reference method for determining measured EE in critically ill patients by measuring volumes of inspired oxygen (VO_2_) and expired carbon dioxide (VCO_2_) [11]. Until recently, indirect calorimetry had not been used in patients receiving ECMO due to the gas exchange occurring at the extracorporeal membrane that is not captured by traditional indirect calorimetry [4]. A novel method of measuring EE has been proposed in patients receiving VV ECMO and has been reported to be feasible, using traditional indirect calorimetry in combination with the calculation of VO_2_ and VCO_2_ from pre- and post- ECMO membrane blood gas analyses [6]. However, this method has not been applied in patients receiving VA ECMO where EE may differ.

The primary objective of our study was to assess the feasibility of measuring EE in patients receiving VA ECMO using a modified indirect calorimetry protocol. Secondary objectives were to report measured EE in patients receiving VA ECMO and to compare values with predictive equation estimates and to control critically ill patients not receiving ECMO.

## Subjects and methods

A single-centre prospective observational study was conducted at The Alfred Intensive Care Unit (ICU) in Melbourne, Australia between 20 August 2019 and 8 September 2021. The Alfred ICU is a quaternary referral centre providing regional services for ECMO. The study was prospectively registered on the Australian New Zealand Clinical Trials Registry (ACTRN12619000760178). Ethics approval was granted from the Alfred Hospital Ethics Committee (project number: 76/19) with informed consent obtained from the medical treatment decision maker. Ethics approval included permission to access a database of indirect calorimetry measurements completed as part of standard practice in the ICU for the comparison of measured EE to control patients not receiving ECMO.

## Eligibility criteria

Patients were considered for inclusion within 72 h of commencement of VA ECMO according to the following criteria:

### Inclusion criteria

- Critically ill adults (≥18 years);
- Commenced on VA ECMO for any reason;
- Expected to require mechanical ventilation for at least 48 h from initiation of ECMO;
- Expected to remain in the ICU and on ECMO for at least 48 h.

### Exclusion criteria

- Patients with a poor prognosis where death was imminent in the subsequent 24 h;
- Where participation in the study was not in the interest of the patient in the opinion of the treating physician.

## Measured energy expenditure

**Fig. 1:**
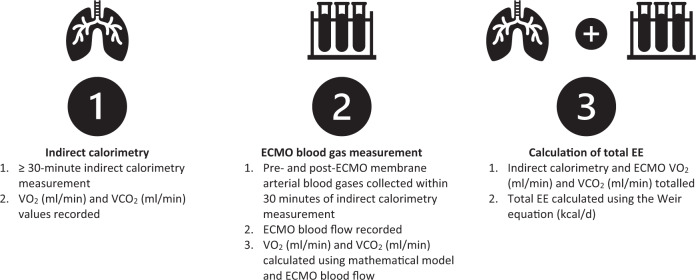
Overview of the modified indirect calorimetry protocol used to measure EE during VA ECMO. ECMO extracorporeal membrane oxygenation, EE energy expenditure, VCO_2_ carbon dioxide production, VO_2_ oxygen consumption.

Measurements of EE were attempted at two timepoints; within 72 h of ECMO commencement (timepoint one [T1]) and on day seven of ICU admission (timepoint two [T2]). If day seven measurements were scheduled on a day of low staff availability (e.g., a weekend), EE measurements were completed prior to and as close to day seven as possible. Energy expenditure was measured as described by Wollersheim et al. in three steps (Fig. 1) [6]. ECMO blood flow, fresh gas flow and ventilator settings were not changed 20 min prior to and during measurement of EE.

### Step (1) Indirect calorimetry

Indirect calorimetry was conducted by trained staff for a minimum of 30 min with the Quark RMR (COSMED, Italy) device while patients were receiving VA ECMO support and were mechanically ventilated. Technical exclusions to measurement included fraction of inspired oxygen (FiO_2_) > 60%, positive end-expiratory pressure (PEEP) > 12 cmH_2_O and known leaks in the ventilator circuit.

### Step (2) blood gas measurements pre- and post- the ECMO membrane

Pre- and post-oxygenator arterial blood gases were collected within 30 min of indirect calorimetry measurement. Samples were collected by an ECMO trained nurse or ECMO credentialed physician from the ECMO pre- and post- oxygenator port. Using a mathematical model published by Dash and Bassingthwaighte, available at www.physiome.org, (model 0149) [12], the O_2_ and CO_2_ content pre- and post- the ECMO membrane were determined using MATLAB version R2019b, Mathworks. In two patients with a low CO_2_ exchange at the oxygenator where the mathematical model generated small negative values of CO_2_ uptake, these values were recorded as zero for physiological plausibility.

### Step (3) Calculation of EE

Values of VO_2_ and VCO_2_ from indirect calorimetry and blood gas measurement were totalled and EE was calculated using the Weir equation [13].

## Clinical and nutrition data collection

Admission and discharge data collected included: Acute Physiology and Chronic Health Evaluation (APACHE) III diagnosis code, APACHE II and III scores, ICU and hospital length of stay and in-hospital mortality. Patients received standard nutrition therapy as per evidence-based feeding protocols used in the unit, with EE estimated by dietitians using the Schofield equation multiplied by a clinically relevant stress factor as per standard practice at The Alfred Hospital. Weight, height, estimated EE and protein requirements were collected from the dietitian notes in the electronic medical record. An adjusted weight was used to calculate requirements in patients classified as overweight or obese. Common practice within the unit is to use a body mass index of 25 kg/m^2^ + 25% excess weight. Estimated EE using the weight-based predictive equation 25 kcal/kg/d, which is commonly recommended and used in the ICU, was also calculated by investigators [6, 14, 15].

## Energy expenditure in control critically ill patients not receiving VA ECMO

Energy expenditure measurements in patients receiving VA ECMO were compared to control critically ill patients not receiving ECMO. Data for controls was sourced from a database of indirect calorimetry measurements completed between January 2010 to July 2019 and stored in the Nutrition Department at The Alfred (manuscript in preparation). Control patients were selected according to APACHE III diagnosis codes and day of indirect calorimetry measurement. One EE measurement was included in analysis per patient as limited repeat indirect calorimetry measurements were available for patients receiving VA ECMO and critically ill control patients.

## Outcomes

### Primary

Feasibility of measuring EE at T1.

### Secondary

1. Feasibility of measuring EE at T2.
2. Difference between EE at T1 and T2.
3. Difference between measured and predicted (Schofield and 25 kcal/kg/d) EE at T1 and T2.
4. Difference between measured EE in patients receiving VA ECMO versus control critically ill patients not receiving ECMO.

## Statistical analyses

The aim was to recruit a convenience sample of 30 mechanically ventilated patients receiving VA ECMO. All data are presented using medians [interquartile range (IQR)]. In the absence of previously published data, feasibility was defined as completion of ≥60% EE measurements at each timepoint. Measured EE was compared between T1 and T2 and to predictive equation estimates using the Wilcoxon signed-rank test. Comparisons between patients receiving VA ECMO and control critically ill patients not receiving ECMO were made using the Mann–Whitney *U* test for continuous variables and the Chi-Square Test for categorical variables. All analyses were performed using IBM® SPSS®, Version 28 (Armonk, NY: IBM Corp) and a *p*-value of <0.05 was considered statistically significant.

## Results

**Fig. 2:**
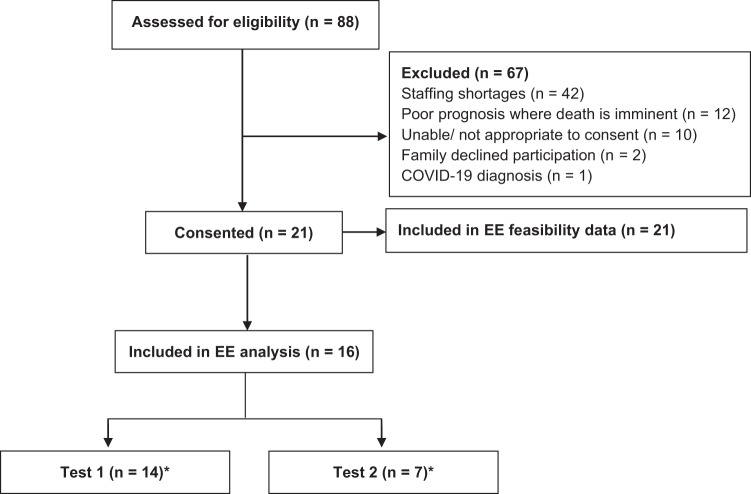
Flow chart of participant recruitment. *n = 5 had measurements at both timepoints. EE energy expenditure.

Over the recruitment period, 21 patients receiving VA ECMO support were included (Fig. 2). The study was stopped prior to meeting the target sample size due to difficulties with recruitment related to the COVID-19 pandemic. All patients were included for the analysis of feasibility while 16 patients had indirect calorimetry measurements for the analysis of measured EE (Fig. 2). Patient demographics are presented in Table 1.

**Table 1:** Demographic and clinical data for all patients receiving VA ECMO (*n* = 21) and those included in energy expenditure analysis (*n* = 16).

Variable	VA ECMO (*n* = 21)	VA ECMO (*n* = 16)
Sex, male, *n* (%)	16 (76)	12 (75)
Age, years	55 [42–64]	53 [40–62]
Weight, kg*	71 [68–109]	74 [69–91]
Height, m*	1.73 [1.66–1.89]	1.75 [1.66–1.82]
BMI, kg/m^2^*	25 [23–29]	25 [23–30]
APACHE II	22 [17–28]	24 [18–28]
APACHE III	83 [71–111]	89 [72–118]
APACHE III diagnosis code, *n* (%):		
Cardiogenic shock	8 (38)	7 (44)
Cardiac arrest	5 (24)	4 (25)
Other cardiovascular disease	3 (14)	3 (19)
Coronary bypass graft	2 (10)	0 (0)
Dissecting aortic aneurysm	1 (5)	1 (6)
Sepsis with shock, other than urinary	1 (5)	1 (6)
Other respiratory diseases	1 (5)	0 (0)
ICU LOS, days	12 [7–25]	17 [7–32]
Hospital LOS, days	20 [9–39]	24 [8–50]
ICU mortality, *n* (%)	7 (33)	5 (31)
Hospital mortality, *n* (%)	7 (33)	5 (31)

Continuous variables are reported as median [IQR].

*n = 20

*APACHE* acute physiology and chronic health evaluation, *BMI* body mass index, *ICU* Intensive Care Unit, *IQR* interquartile range, *LOS* length of stay.

## Feasibility of completing energy expenditure measurements

**Fig. 3:**
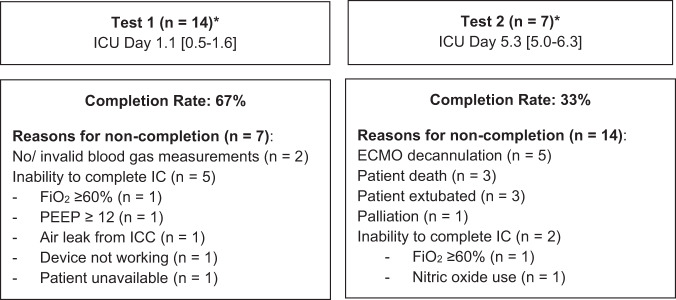
Feasibility of completing energy expenditure measurements in patients receiving VA ECMO. *n = 5 had measurements at both timepoints. ECMO extracorporeal membrane oxygenation, FiO_2_ fraction of inspired oxygen, IC indirect calorimetry, ICC intercostal catheter, PEEP positive end-expiratory pressure.

In total, 21 EE measurements were conducted during the study period. Energy expenditure was measured in 14 of the 21 patients (67%) at T1 on ICU day 1 [1, 2]. Reasons for non-completion are outlined in Fig. 3, with contraindications to indirect calorimetry the most common reason. Energy expenditure was measured in 7 of the 21 patients (33%) at T2 on ICU day 5 [5, 6], with the most common reason for test non-completion being ECMO decannulation (Fig. 3).

## Measured energy expenditure

All patients were afebrile at the time of EE measurement and were receiving continuous enteral nutrition. Overall, EE was 1570 [1328–1879] kcal/d (*n* = 21); 1454 [1213–1860] kcal/d at T1 (*n* = 14) and 1657 [1570–2074] kcal/d at T2 (*n* = 7), *P* = 0.043. Measured EE was equivalent to 22 [15–24] kcal/kg/d at T1 and 25 [21–26] kcal/kg/d at T2. Table 2 outlines the VO_2_ and VCO_2_ contribution of indirect calorimetry and ECMO blood gas measurement to total EE.

**Table 2:** Contribution of pulmonary indirect calorimetry and ECMO to total EE (*n* = 21).

Variable	Indirect calorimetry	ECMO blood gas measurement	Indirect calorimetry + ECMO blood gas measurement
VO_2_, ml/min	119 [55–160]	105 [74–148]	224 [186–243]
VCO_2_, ml/min	83 [36–121]	119 [61–218]	217 [145–320]
EE, kcal/d			1570 [1328–1879]

Continuous variables are reported as median [IQR].

*EE* energy expenditure, *ECMO* extracorporeal membrane oxygenation.

## Measured energy expenditure versus predictive equation estimates

**Fig. 4:**
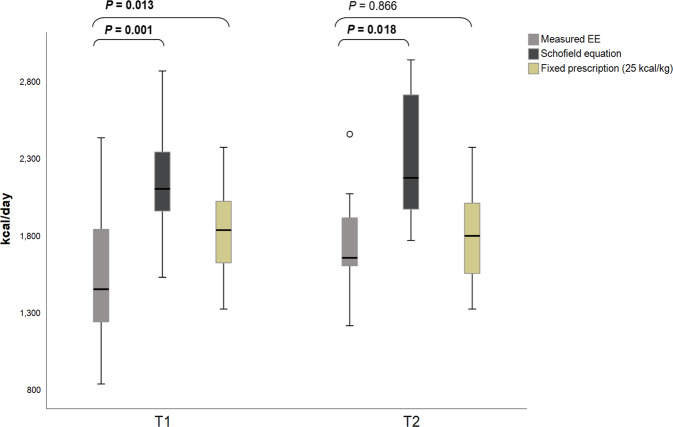
Measured versus Estimated EE. Comparison of measured versus estimated EE using the Schofield and 25 kcal/kg/d predictive equations in kcal/d.

The Schofield predictive equation was used to estimate EE in all patients with a stress factor of 1.3 [1.2–1.3] applied at T1 and 1.5 [1.4–1.5] at T2 as determined by the treating dietitian. Estimated EE using the Schofield equation was 2105 [1950–2452] kcal/d overall; 2105 [1878–2345] at T1 (*n* = 14) and 2177 [1938–2871] kcal/d at T2 (*n* = 7). Estimated EE using 25 kcal/kg/d was 1800 [1563–2025] kcal/d overall; 1837 [1594–2056] at T1 (*n* = 14) and 1800 [1488–2025] kcal/d at T2 (*n* = 7). The Schofield equation with stress factor overestimated EE at both timepoints while the 25 kcal/kg/d equation overestimated EE at T1 but was comparable to EE at T2 (Fig. 4).

## Measured energy expenditure in patients receiving VA ECMO versus control critically ill patients

There were no differences in patient characteristics or the day of indirect calorimetry measurement between patients receiving VA ECMO and control critically ill patients (*n* = 16) (Table S1). Measured EE was lower in patients receiving VA ECMO versus control critically ill patients but the difference was not statistically significant; 1577 [1434–1801] versus 2092 [1609–2272] kcal/d, respectively (*P* = 0.056).

## Discussion

In this study which is the largest report of measured EE in patients receiving VA ECMO, we found that a modified indirect calorimetry protocol was feasible to apply early in ICU admission to measure EE but not later in admission. Measurements of EE increased during the first week of ICU admission but were lower than predictive equation estimates and the EE of a cohort of control critically ill patients not receiving ECMO support.

Although found to be feasible early in ICU admission, EE measurements were only completed in approximately two-thirds of patients on day one of ICU admission. Conversely, EE measurements were only possible in one-third of patients on day five of ICU admission and were not considered feasible to complete. Reasons for non-completion on day one were mainly related to the inability to complete indirect calorimetry measurements due to contraindications (e.g. PEEP and FiO_2_ levels above manufacturer specifications) and on day five were due to ECMO decannulation, extubation or death. Measurements of EE were not attempted following ECMO decannulation in our study which should be considered when interpreting feasibility results. Similar findings were reported by Wollersheim et al. where EE was measured in 20 of 24 patients within two days of ECMO commencement and 8 of 24 patients directly before and after VV ECMO decannulation [6].

Measurements of EE were lower on day one compared to day five of ICU admission likely reflecting the Ebb phase of the metabolic response to critical illness, which is marked by decreases in core temperature, basal metabolic rate and cardiac output in the first 24–48 h of ICU admission [16]. Values for EE were lower than predicted by the unit dietitian using the Schofield equation multiplied by stress factor at both timepoints. Conversely, the weight based 25 kcal/kg/d equation overestimated EE on day one but was comparable to values on day five of ICU admission. This is an interesting finding as predictive equations have been shown to be more prone to underestimation, rather than overestimation, of EE in general ICU populations [9, 17]. Further, EE was also lower than control critically ill patients not receiving ECMO support, albeit this difference did not reach statistical significance.

Previously, it was speculated that use of ECMO was associated with increased inflammation and subsequent hypermetabolism [5]. Recent studies have reported decreases in inflammation following commencement of VV ECMO and EE comparable to other critically ill subpopulations [6, 7]. Although using different methods, our findings are comparable to De Waele et al. where indirect calorimetry was used to measure EE at both the ventilator and at the ECMO membrane oxygenator. In this study, a median EE of 1334 kcal/d equivalent to 21 kcal/kg/d was reported in seven patients receiving VV (*n* = 3) and VA ECMO (*n* = 4) [15]. In contrast, Wollersheim et al reported a higher median EE of 2013 kcal/d in 20 patients with acute respiratory distress syndrome receiving VV ECMO [6] and Ong et al. reported a mean EE of 29 ± 6 kcal/kg/d in seven patients treated with VV ECMO when not prescribed paralytic agents [18]. However, the latter study determined EE at one timepoint using blood gas measurements pre- and post- the ECMO membrane and mathematical equations to calculate VO_2_ and VCO_2_, without completion of indirect calorimetry [18]. Reasons for the higher EE observed by Wollersheim et al. and Ong et al. may be due to the type of ECMO (VA versus VV) and admission diagnosis (predominantly cardiogenic shock in our study versus acute respiratory distress syndrome in Wollersheim et al.). The use of VA ECMO provides both respiratory and haemodynamic support, replacing the function of the heart and lungs, which may be associated with lower EE. This relief of cardiac work may similarly explain why EE was lower in patients receiving VA ECMO compared to control critically ill patients in our study. While severity of illness was comparable between groups, data was not collected on the use of sedatives, paralytic agents, vasopressors or analgesics at the time of measurement, which may have also contributed to differences in EE.

### Strengths and limitations

This study was registered a priori and provides the largest dataset of measured EE in patients receiving VA ECMO, which is particularly important for centres that are not able to perform indirect calorimetry but care for critically ill patients receiving ECMO. The assessment of EE was hampered by research staff unavailability during the COVID 19 pandemic and this is a limitation. The sample size and single-centre design should be considered when interpreting generalisability of findings. However, it does approximate the size of other similar studies and combined, all studies provide valuable information. Data for control patients was sourced from a database of indirect calorimetry measurements where temperature at the time of EE measurement was not routinely collected. The model by Dash and Bassingthwaighte is mathematically sound and has been validated but not in the context of an extracorporeal oxygenator. Small margins of measurement errors of pre- and post- the oxygenator gas may occur; it is unknown how this compares to volumetric gas measurements. Ideally new methods would be validated prior to use to ensure accuracy; however, validation of methods involving metabolism are technically difficult in the acute setting [19].

### Clinical implications and future research

The clinical implications of our work are that measured EE may be lower in patients receiving VA ECMO early in ICU admission than initially hypothesised, a finding that is supported by other work [15]. Clinicians should be aware of the possibility of overestimation of EE with predictive equations early in illness. However, standard care delivery of nutrition often only approximates 50–60% of prescribed volumes in critical illness and this should also be a consideration to prevent the profound under provision of nutrition in a patient group which is likely to have prolonged ICU and hospital admissions [10, 20, 21]. This reinforces the importance of indirect calorimetry to enable tailored nutrition provision and align management with best practice guidelines. Future work should explore the accuracy of the present method and other techniques of measuring gas exchange at the oxygenator to continue to test the practicability of using indirect calorimetry to measure temporal changes in EE. Differences in ECMO support modality and underlying diagnoses in these severely ill patient groups may exist and need to be explored on a larger scale.

In conclusion, it is feasible to use a modified indirect calorimetry protocol to measure EE in patients receiving VA ECMO early in ICU admission, but this may not be possible in all patients, especially later in admission. Energy expenditure may be lower in critically ill patients receiving VA ECMO compared to other critically ill populations and predictive estimates may be more prone to overestimation of EE.

## Supplementary information

Table S1

## Data Availability

Requests for the datasets generated during and/or analysed will be considered based on reasonable request to the corresponding author.

## References

[1] Brain, MJ, Butt, WW, MacLaren, G (2022). Physiology of Extracorporeal Life Support (ECLS). In: Schmidt, G.A. (eds.) Extracorporeal membrane oxygenation for adults. Respiratory Medicine. Humana, Cham. 10.1007/978-3-031-05299-6_1

[2] Park J, Heo E, Song IA, Cho J, Namgung H, Lee E (2020). Nutritional support and clinical outcomes in critically ill patients supported with veno-arterial extracorporeal membrane oxygenation. Clin Nutr.

[3] MacGowan L, Smith E, Elliott-Hammond C, Sanderson B, Ong D, Daly K (2019). Adequacy of nutrition support during extracorporeal membrane oxygenation. Clin Nutr.

[4] Kagan I, Singer P (2013). Nutritional imbalances during extracorporeal life support. World Rev Nutr Diet.

[5] Wollersheim, T, Müller, MC, Weber-Carstens, S (2018). ECMO patients. In: Berger, M (ed.). Critical care nutrition therapy for non-nutritionists. Springer, Cham. 10.1007/978-3-319-58652-6_3

[6] Wollersheim T, Frank S, Müller MC, Skrypnikov V, Carbon NM, Pickerodt PA (2018). Measuring energy expenditure in extracorporeal lung support patients (MEEP) - Protocol, feasibility and pilot trial. Clin Nutr.

[7] Burrell AJC, Lubnow M, Enger TB, Nanjayya VB, Philipp A, Malfertheiner MV (2017). The impact of venovenous extracorporeal membrane oxygenation on cytokine levels in patients with severe acute respiratory distress syndrome: a prospective, observational study. Crit Care Resusc.

[8] Chung M, Shiloh AL, Carlese A (2014). Monitoring of the adult patient on venoarterial extracorporeal membrane oxygenation. The Scientific World Journal.

[9] Tatucu-Babet OA, Ridley EJ, Tierney AC (2016). Prevalence of underprescription or overprescription of energy needs in critically Ill mechanically ventilated adults as determined by indirect calorimetry: a systematic literature review. JPEN J Parenter Enter Nutr.

[10] Oude Lansink-Hartgring A, van Minnen O, Vermeulen KM, van den Bergh WM, Oude Lansink-Hartgring A, van den Bergh WM (2021). Hospital costs of extracorporeal membrane oxygenation in adults: a systematic review. PharmacoEconomics - Open.

[11] Singer P, Blaser AR, Berger MM, Alhazzani W, Calder PC, Casaer MP (2019). ESPEN guideline on clinical nutrition in the intensive care unit. Clin Nutr.

[12] Dash RK, Bassingthwaighte JB (2010). Erratum to: blood HbO2 and HbCO2 dissociation curves at varied O2, CO2, pH, 2,3-DPG and temperature levels. Ann Biomed Eng.

[13] Weir JB (1949). New methods for calculating metabolic rate with special reference to protein metabolism. J Physiol.

[14] Dresen E, Naidoo O, Hill A, Elke G, Lindner M, Jonckheer J (2023). Medical nutrition therapy in patients receiving ECMO: evidence-based guidance for clinical practice. JPEN J Parenter Enter Nutr.

[15] De Waele E, Jonckheer J, Pen JJ, Demol J, Staessens K, Puis L (2019). Energy expenditure of patients on ECMO: a prospective pilot study. Acta Anaesthesiol Scand.

[16] Hill AG, Hill GL (1998). Metabolic response to severe injury. Br J Surg.

[17] Kross EK, Sena M, Schmidt K, Stapleton RD (2012). A comparison of predictive equations of energy expenditure and measured energy expenditure in critically ill patients. J Crit Care.

[18] Ong CS, Brown P, Shou BL, Wilcox C, Cho S-M, Mendez-Tellez PA (2022). Resting energy expenditure of patients on venovenous extracorporeal membrane oxygenation for adult respiratory distress syndrome: a pilot study. Crit Care Explor.

[19] Tatucu-Babet OA, Nguo K, Lambell KJ, Romero L, Earthman CP, Ridley EJ (2022). Doubly labelled water for determining total energy expenditure in adult critically ill and acute care hospitalized inpatients: a scoping review. Clin Nutr.

[20] Ridley EJ, Peake SL, Jarvis M, Deane AM, Lange K, Davies AR (2018). Nutrition therapy in Australia and New Zealand intensive care units: an international comparison study. JPEN J Parenter Enter Nutr.

[21] Ridley EJ, Davies AR, Robins EJ, Lukas G, Bailey MJ, Fraser JF (2015). Nutrition therapy in adult patients receiving extracorporeal membrane oxygenation: a prospective, multicentre, observational study. Crit Care Resusc.

