# Supplementary material for: Modified indirect calorimetry for patients on venoarterial extracorporeal membrane oxygenation: a pilot feasibility study
Source: Eur J Clin Nutr. 2023 May 17;77(9):888–94. doi: 10.1038/s41430-023-01291-x (PMC10191396; doi:10.1038/s41430-023-01291-x)
Supplement: Supplementary file 1 — Table S1 [file 41430_2023_1291_MOESM1_ESM.pdf]

Table S1. Demographic and clinical data for patients receiving VA ECMO versus control critically ill patients

Variable	VA ECMO (n = 16)	Non-ECMO (n = 16)	p-values
Sex, male, n (%)	12 (75)	12 (75)	1.00
Age, years	53 [40-62]	56.3 [48-64]	0.27
Weight, kg	74 [69-91]	85 [70-100]	0.34
Height, m	1.75 [1.66-1.82]	1.77 [1.70-1.82]	0.64
BMI, kg/m^2	25 [23-30]	26 [25-37]	0.47
APACHE II	23.5 [17.8-28.0]	23.0 [16-26]	0.34
APACHE III	89.0 [71.5-118.3]	76.0 [49-109]	0.27
APACHE III diagnosis code, n (%):			0.81
Cardiogenic shock	7 (44)	6 (38)	
Cardiac arrest	4 (25)	4 (25)	
Other cardiovascular disease	3 (19)	5 (31)	
Sepsis with shock, other than urinary	1 (6)	1 (6)	
Dissecting aortic aneurysm	1 (6)	0 (0)	
ICU LOS, days	17 [7-32]	9 [7-16]	0.27
Hospital LOS, days	24 [8-50]	28 [10-59]	0.67
ICU mortality, n (%)	5 (31)	4 (25)	0.69
Hospital mortality, n (%)	5 (31)	4 (25)	0.69

Continuous variables are reported as median [IQR].

Abbreviations: APACHE, acute physiology and chronic health evaluation; BMI, body mass index; ICU, Intensive Care Unit; IQR, interquartile range; LOS, length of stay.
